# Research progress on branched-chain amino acid aminotransferases

**DOI:** 10.3389/fgene.2023.1233669

**Published:** 2023-11-06

**Authors:** Can Chen, Hassan Naveed, Keping Chen

**Affiliations:** ^1^ School of Life Sciences, Jiangsu University, Zhenjiang, China; ^2^ School of Food and Biological Engineering, Jiangsu University, Zhenjiang, China

**Keywords:** branched-chain amino acid aminotransferases, branched-chain amino acids, evolutionary tree, metabolism, human disease

## Abstract

Branched-chain amino acid aminotransferases, widely present in natural organisms, catalyze bidirectional amino transfer between branched-chain amino acids and branched-chain α-ketoacids in cells. Branched-chain amino acid aminotransferases play an important role in the metabolism of branched-chain amino acids. In this paper, the interspecific evolution and biological characteristics of branched-chain amino acid aminotransferases are introduced, the related research of branched-chain amino acid aminotransferases in animals, plants, microorganisms and humans is summarized and the molecular mechanism of branched-chain amino acid aminotransferase is analyzed. It has been found that branched-chain amino acid metabolism disorders are closely related to various diseases in humans and animals and plants, such as diabetes, cardiovascular diseases, brain diseases, neurological diseases and cancer. In particular, branched-chain amino acid aminotransferases play an important role in the development of various tumors. Branched-chain amino acid aminotransferases have been used as potential targets for various cancers. This article reviews the research on branched-chain amino acid aminotransferases, aiming to provide a reference for clinical research on targeted therapy for various diseases and different cancers.

## 1 Introduction

**FIGURE 1:**
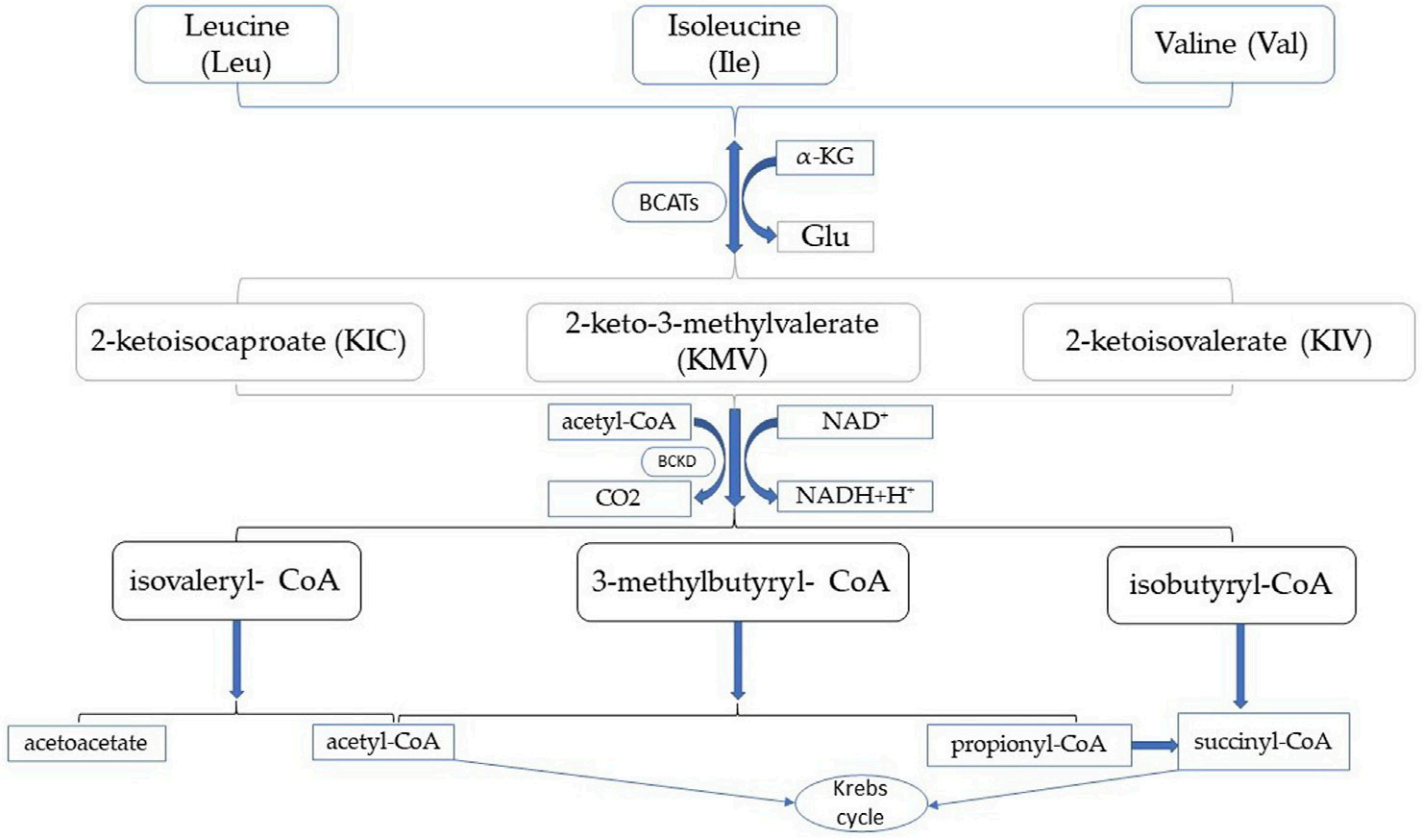
Branched-chain amino acid metabolism.

Branched-chain amino acid aminotransferases (BCATs) play a vital role in the metabolism of branched-chain amino acids (BCAAs) and are an important regulator. BCAT is the only enzyme common to BCAA biosynthesis and degradation and BCAT activity affects BCAA homeostasis. BCAAs include valine (Val), leucine (Leu), and isoleucine (Ile) (Shou et al., 2019). Humans cannot synthesize branched-chain amino acids, and they need to be ingested from the outside through diet. BCAAs play an important role, with leucine known for its vital role in protein anabolism by activating the mammalian target of rapamycin (mTOR) signaling pathway (Dimou et al., 2022). BCAAs play an important role in skeletal muscle as an energy source. During exercise, BCAAs, especially leucine, transaminated in skeletal muscle, producing acetyl-CoA to the Krebs cycle. This amino group can be transamimoniated to alanine, which is produced in the liver through the glucose-alanine cycle, producing excess liver glycogen (de Campos-Ferraz et al., 2014). In addition to the effects on energy expenditure in exercise as well as muscle damage, proper BCAA supplementation may have relatively beneficial effects on many pathologies, such as liver and kidney disease (Dimou et al., 2022) and muscle wasting disorders (Cano et al., 2006). It can also act as an important nutritional signal and metabolic regulator and has a role in glucose homeostasis, the nervous system, and the immune response (Holeček, 2018). BCAAs metabolism differs from other essential amino acid metabolism. BCAAs are first transported in extrahepatic tissues and need to be shuttled between organs or tissues for complete catabolism (Ananieva and Wilkinson, 2018). The first step in human BCAA catabolism is the reversible transamination process, BCAAs transfer amino groups to α-ketoglutarate (α-KG) to produce 2-ketoisocaproate (KIC), 2-keto-3-methylvalerate (KMV), 2-ketoisovalerate (KIV), and glutamic acid (Glu). Since BCAT catalyzes the reaction is reversible, the active center of BCAT is the recognition of acidic amino acids (Glu) as well as hydrophobic amino acids (BCAAs), a dual substrate recognition mechanism (Goto et al., 2003). BCAT activity is high in skeletal muscle (Shimomura et al., 2006). Therefore, it is thought that this step is mainly performed in skeletal muscle. The second step is catalyzed by Branched chain α-ketoacid dehydrogenase complex, it is located in the inner mitochondrial membrane and irreversibly oxidizes α-ketoacids to produce isovaleryl-CoA, 3-methylbutyryl- CoA, isobutyryl-CoA, CO2 and NADH. BCKD kinase (BCKDK) phosphorylates BCKD leading to its inactivation, protein phosphatase 2Cm (PP2Cm) dephosphorylates BCKD leading to its activation, and the activity of BCKDC is strictly regulated to maintain BCAA homeostasis *in vivo* (Islam et al., 2010). The third step of BCAA catabolism is the production of acetyl-CoA and succinyl-CoA into the Krebs cycle, which produces ATP, as shown in Figure 1. In contrast to other tissues, the BCAA degradation process occurs mainly in muscles and the liver. BCAA catabolism involves two initial enzymatic reactions common to all BCAAs, and the intake of a single BCAA affects the catabolism of all three BCAAs (Hutson et al., 2005).

BCAT participates in metabolic reactions, is closely related to some animal and plant diseases, and participates in plant defense responses and fruit ripening. BCAT is also closely related to human conditions, such as diabetes, neurological diseases, etc. Decreased glycolysis in diabetic patients leads to decreased aminoreceptors (α-ketoglutarate), and decreased transamination of branched-chain amino acids leads to accumulation of branched-chain amino acids (Holeček, 2020). Studies have found a positive correlation between severity and commonplace BCAT levels in Alzheimer’s patients (Hudd et al., 2019), and demonstrates a high expression in many types of tumors, having an obvious correlation with the value-added invasion and prognosis of tumor cells. Earlier studies have focused on characterizing the structure of BCAT, analyzing the relationships between enzymes and substrates and the biological properties of enzymes, and cloning expression purification, which will facilitate the industrial production of enantiomeric chiral amino acids by aminotransferases. With the rapid development of scientific and technological methods, more attention is paid to research at the molecular level, the expression signaling pathway of the BCAT gene, and the relationship between BCAT and disease. The latter is analyzed more comprehensively and specifically by combining the genomic transcriptome and metabolomics.

## 2 Evolution and biological characteristics of branched-chain amino acid aminotransferases

BCAA aminotransferases are classified as folded type IV aminotransferases, a pyridoxal 5′-phosphate (PLP)-dependent enzyme (Schiroli and Peracchi, 2015). They play a vital role in living organisms. With the rapid development of genome sequencing technology, the protein gene sequences are becoming clearer. The BCAT gene sequences of different species can be obtained, and the differences among species can be understood at the molecular level. The NCBI website has recorded the genetic sequences of branched-chain amino acid aminotransferases from more than 300,000 species, and a variety of BCAT protein sequences have been described.

For most eukaryotes, including humans, there are two subtypes of branched-chain amino acid aminotransferases: BCAT1 and BCAT2 (Papathanassiu et al., 2017). It has long been thought that these two enzymes have similar physiological functions in cells and drive the same transamination response. But studies have gradually revealed differences in their physiological functions and regulatory mechanisms (Toyokawa et al., 2021). BCAT1 encodes a cytoplasmic protein expressed primarily in the brain that provides nitrogen for cerebral glutamate synthesis (Wang et al., 2015) and is secondarily expressed in embryonic tissue, the ovaries, the placenta, and neurons of the peripheral nervous system, regulating mammalian target of rapamycin complex 1 (mTORC1) signaling and glycolytic metabolism in CD4^+^ T cells (Ananieva et al., 2014). BCAT2 encodes a mitochondrial protein commonly expressed in almost all organs (Knerr et al., 2019).

*BCATs* have been identified in plants such as Arabidopsis, rice, wheat, rape, tomato, etc., which are mostly cash crops. As a representative model plant, *Arabidopsis thaliana* has been extensively studied in many aspects, and six BCAT subtypes have been identified in *Arabidopsis* (Diebold et al., 2002). Six BCAT genes (SIBCAT) were identified in tomatoes (Maloney et al., 2010). SlBCAT1, SlBCAT2, SlBCAT3, and SlBCAT4 are expressed in a variety of plant tissues. While SlBCAT5 and SlBCAT6 are undetectable, six isoforms are active in both forward (BCAA synthesis) and reverse (branched-chain ketoacid synthesis) reactions. Detailed BCAT information is shown in Table 1. Different subtypes of BCAT perform different functions in different subunits of cells, and the enzyme kinetic characteristics are different (Km values are shown in Table 2), the smaller the Km value, the greater the enzyme affinity for substrates, which verifies that mitochondrial SlBCAT1 and SlBCAT2 play a role in BCAA catabolism, while chloroplasts SlBCAT3 and SlBCAT4 play a role in BCAA synthesis (Maloney et al., 2010). Comparing the kinetic parameters of enzymes in different species or different subtypes of the same species can help better understand the function of enzymes and the differences in function.

**TABLE 1:** Kenetic parameters of BCATs.

Km value (mM)	Kcat/Km value [1/mMs-1]	Substrate	Organism	Transaminase isoforms and reaction conditions	References
0.56	70	L-leucine	*Solanum lycopersicum*	SlBCAT1, pH not specified, 25°C	Maloney et al. (2010)
0.2	40			SlBCAT2, pH not specified, 25°C	
2.7	45			SlBCAT3, pH not specified, 25°C	
0.57	31			SlBCAT4, pH not specified, 25°C	
1.8	66			SlBCAT5, pH not specified, 25°C	
0.21	75			SlBCAT6, pH not specified, 25°C	
0.42	−		*Escherichia coli*	pH 8.0, 25°C	Bezsudnova et al. (2017)
0.62	−		*Homo sapiens*	pH 8.4, 25°C, 2-oxoglutarate as amino group acceptor	Schadewaldt (2000)
0.67	61	L-isoleucine	*Solanum lycopersicum*	SlBCAT1, pH not specified, 25°C	Maloney et al. (2010)
0.31	25			SlBCAT2, pH not specified, 25°C	
4.9	36			SlBCAT3, pH not specified, 25°C	
0.43	47			SlBCAT4, pH not specified, 25°C	
3.2	51			SlBCAT5, pH not specified, 25°C	
0.36	59			SlBCAT6, pH not specified, 25°C	
0.52	−		*Escherichia coli*	pH 8.0, 37°C	Lee-Peng et al. (1979)
10.3	−		*Homo sapiens*	pH 8.0, 37°C, isoenzyme I, 2-oxoglutarate as amino group acceptor	Kido (1988)
3	−			pH 8.0, 37°C, isoenzyme III, 2-oxoglutarate as amino group acceptor	Kido (1988)
1	50	L-valine	*Solanum lycopersicum*	SlBCAT1, pH not specified, 25°C	Maloney et al. (2010)
1.4	3			SlBCAT2, pH not specified, 25 °C	
2	56			SlBCAT3, pH not specified, 25°C	
1.4	15			SlBCAT4, pH not specified, 25°C	
2.6	47			SlBCAT5, pH not specified, 25°C	
1.2	20			SlBCAT6, pH not specified, 25°C	
2.7	−		*Escherichia coli*	pH 8.0, 25°C	Inoue et al. (1988)
2.96	−		*Homo sapiens*	pH 8.4, 25°C, 2-oxoglutarate as amino group acceptor	Schadewaldt (2000)

**TABLE 2:** Characteristics of BCATs.

Name	Transaminase subtype	Location in the cell	Characteristics	References
*Homo sapiens*	BCAT1	cytoplasm	Expressed mainly in the brain	Wang et al. (2015)
	BCAT2	mitochondria	Expressed in almost all organs	Knerr et al. (2019)
*Oryza sativa*	OsDIAT	cytoplasm	Regulate drought resistance	Shim et al. (2023)
*Solanum lycopersicum*	SlBCAT1	mitochondria	High branched-chain ketoacid synthesis activity, multi-plant tissue expression	Maloney et al. (2010)
	SlBCAT2	mitochondria	High branched-chain ketoacid synthesis activity, multi-plant tissue expression	Maloney et al. (2010)
	SlBCAT3	chloroplast	Role in BCAA synthesis, multi-plant tissue expression	Maloney et al. (2010)
	SlBCAT4	chloroplast	Role in BCAA synthesis, multi-plant tissue expression	Maloney et al. (2010)
	SlBCAT5	cytoplasm	Undetectable	Maloney et al. (2010)
	SlBCAT6	vacuole	Undetectable	Maloney et al. (2010)
*Arabidopsis thaliana*	AtBCAT1	mitochondria	It is mainly active in catabolism	Diebold et al. (2002)
	AtBCAT2	chloroplast	Acts in BCAA synthesis and is expressed only in flowers	Diebold et al. (2002)
	AtBCAT3	chloroplast	Role in BCAA synthesis	Diebold et al. (2002)
	AtBCAT4	cytoplasm	Lack of targeting sequences	Diebold et al. (2002)
	AtBCAT5	chloroplast	Role in BCAA synthesis	Diebold et al. (2002)
	AtBCAT6	cytoplasm	Lack of targeting sequence, expressed in flowered carpus	Diebold et al. (2002)
*Triticum aestivum*	Ta BCAT1	mitochondria	Role in BCAA synthesis	Corredor-Moreno et al. (2021)
*Magnaporthe oryzae*	BAT1	mitochondria	Role in BCAA synthesis	Que et al. (2020)
	BAT2	cytoplasm	Deletion of this gene	Que et al. (2020)
	BAT3	——	Postulated	Que et al. (2020)
*Mortierella alpina*	BatA	mitochondria	Role in BCAA synthesis	Sonnabend et al. (2022)
	——	——	——	Sonnabend et al. (2022)
	BatC	——	Homologous to the BAT3 gene	Sonnabend et al. (2022)
*Saccharomyces cerevisiae*	Bat1	mitochondria	Not only in the production of BCAAs but also in the generation of fusel alcohols	Toyokawa et al. (2021)
	Bat2	cytoplasm	Not only in the production of BCAAs but also in the generation of fusel alcohols	Toyokawa et al. (2021)
*Pseudomonas* sp.	PsBCAT	cytoplasm	Activity of aromatic L-amino acids, L-histidine, L-lysine and L-threonine	Zheng et al. (2019)
*Aspergillus nidulans*	BatA	mitochondria	Not necessary for the synthesis of BCAA.	Shimizu et al. (2010)
	BatB	cytoplasm	Not necessary for the synthesis of branched-chain amino acids, mainly catabolic enzymes	Shimizu et al. (2010)
	BatC	mitochondria	Not necessary for the synthesis of BCAA.	Shimizu et al. (2010)
	BatD	cytoplasm	Not necessary for the synthesis of BCAA.	Shimizu et al. (2010)
	BatE	cytoplasm	Not necessary for the synthesis of BCAA.	Shimizu et al. (2010)
	BatF	cytoplasm	Not necessary for the synthesis of BCAA.	Shimizu et al. (2010)

The BCATs in *Saccharomyces cerevisiae* is encoded by two genes, BCAT1 and BCAT2 (Eden et al., 1996). BCAT1 is mainly in mitochondria, while BCAT2 is in the cytoplasm (Kispal et al., 1996). Six genes encoding BCAA aminotransferase were identified in *Aspergillus nidularis*, a filamentous fungus. Six BAT isoenzymes as well as coding genes were identified by using BLAST analysis (Shimizu et al., 2010); details are shown in Table 1. The *S. cerevisiae* BAT protein sequence shows different degrees of similarity compared with the six BAT enzymes of *A. nidularis* (Steyer et al., 2021). Two BCAA aminotransferases (BCATa and BCATb) play a role in Ile and Val biosynthesis and play a major role in Leu biosynthesis. The gene encoding BCAT in *Mycobacterium tuberculosis* is a member of the subfamily IIIa of transaminases. The *M. tuberculosis* IlvE monomer consists of two domains that interact to form the active site, and the conserved N-terminal Phe30 residue can confer additional substrate selectivity (Tremblay and Blanchard, 2009). D- and L-cycloserine can inactivate branched-chain aminotransferases of *M. tuberculosis* and inhibit multiple PLP-dependent enzymes (Amorim Franco et al., 2017). BCAT catalyzes the formation of methionine from α-keto-γ-methiolbutyrate (Venos et al., 2004), and all mycobacterial BCAT sequences were found to be identical to *M. tuberculosis* sequences except for those in *M. bovum* (Venos et al., 2004). BCAT affects the growth and survival of *Mycobacterium tuberculosis*, so it may be a candidate gene for the development of inhibitors (Amorim Franco et al., 2016). *Staphylococcus* can also catalyze the transamination of methionine to a certain extent (Madsen et al., 2002).

**FIGURE 2:**
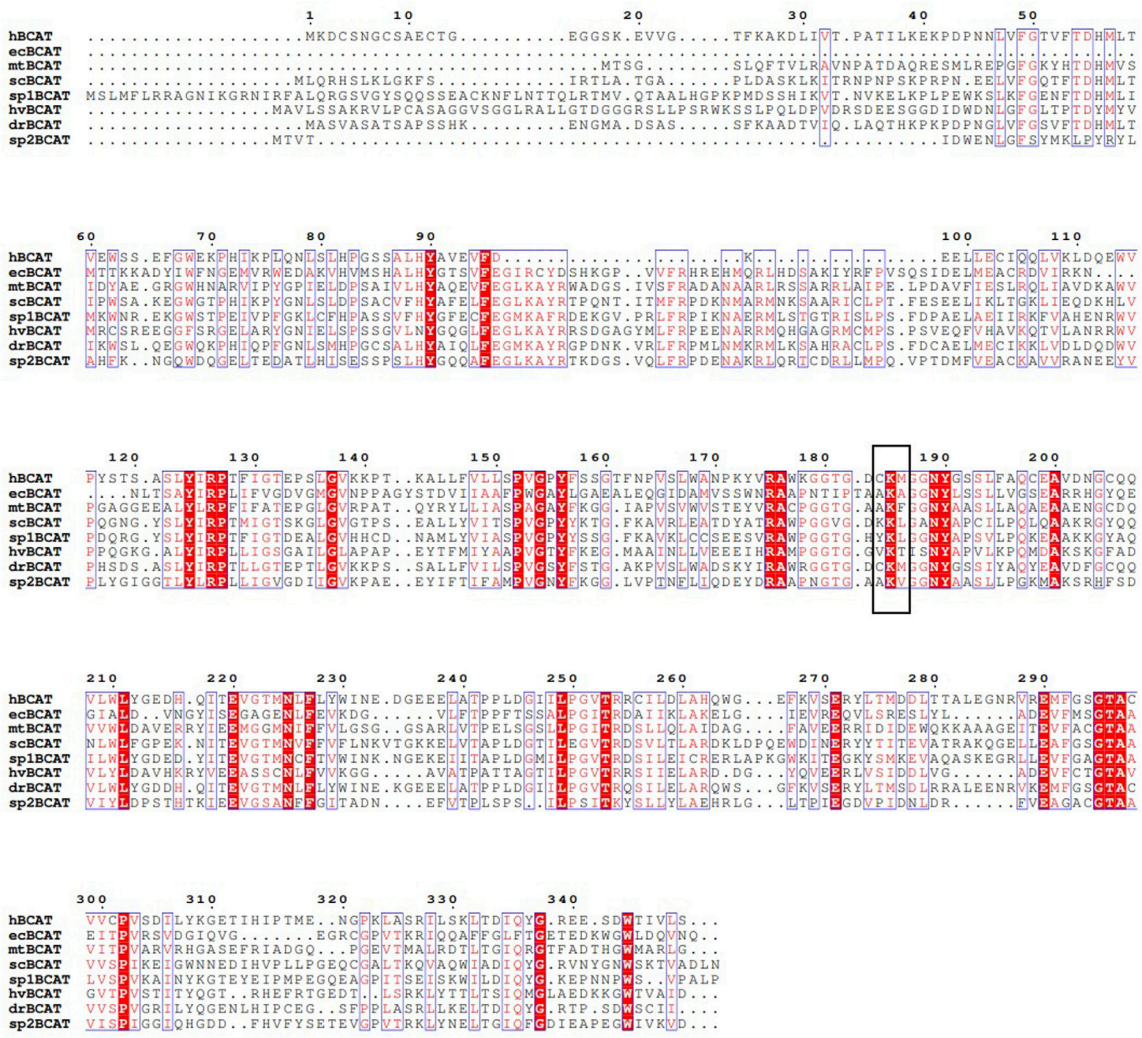
Multiple alignment of *Homo sapiens*, *Escherichia coli, Mycobacterium tuberculosis, Saccharomyces cerevisiae, Schizosaccharomyces pombe, Hordeum vulgare, Darnio rerio, Streptococcus pneumoniae* BCAT amino acid sequence. A black box indicates the conserved-catalytic lysine residues of BCATs. hBCAT, ecBCAT, mtBCAT, scBCAT, sp1BCAT, hvBCAT, drBCAT and sp2BCAT indicate BCATs derived from *Homo sapiens, Escherichia coli, Mycobacterium tuberculosis, Saccharomyces cerevisiae, Schizosaccharomyces pombe, Hordeum vulgare, Darnio rerio, Streptococcus pneumoniae,* respectively.

In order to understand the differences of BCAT in different species, the BCAT of different species was analyzed by primary structure, and the conservatism of BCAT sequences of animals, plants, and microorganisms was analyzed. A multi-sequence alignment analysis of *Homo sapiens, Escherichia coli, Mycobacterium tuberculosis, Saccharomyces cerevisiae, Schizosaccharomyces pombe, Hordeum vulgare, Darnio rerio, Streptococcus pneumoniae* BCAT was conducted, and the results are shown in Figure 2. Understanding the differences in the primary structure of BCAT has a significant impact on enzyme function. BCAT is a PLP-dependent enzyme that binds to conserved catalytic lysine residues (as shown in Figure 2, the black box indicates the conserved catalytic lysine residues of BCATs). In *Saccharomyces cerevisiae* BCATs, amino acid substitution of lysine at position 219 in BCAT1 and position 202 in BCAT2 has been reported to lose its enzymatic catalytic activity (Kingsbury et al., 2015). More importantly, the amino acid substitution of lysine residues catalyzed by BCAT2 (K202H and K202M) not only led to the loss of catalytic activity, but also yeast cells expressed these dysfunctional Bat2 proteins under multiple stress conditions (e.g., pH, caffeine.), showing different growth phenotypes compared to wild-type cells (Espinosa-Cantú et al., 2018). Therefore, understanding the primary structure of the enzyme will give a clearer understanding of the function of the enzyme.

**FIGURE 3:**
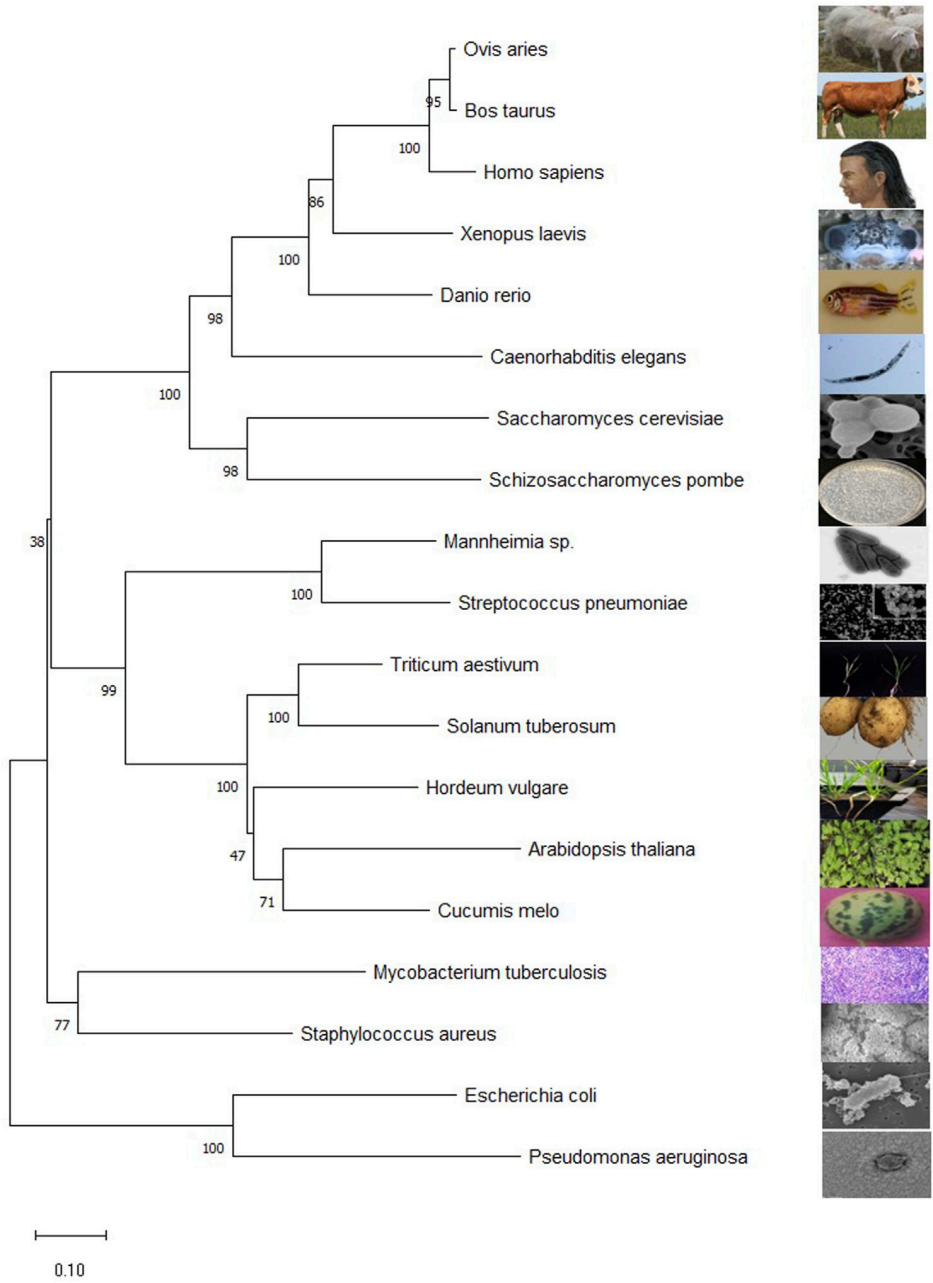
Mega11 maps the *BCAT* gene phylogenetic tree. The sequences for all species are from NCBI. *Homo sapiens* (NP_001171562.1), *Escherichia coli* str. K-12substr.MG1655(YP_026247.1), *A. thaliana* (NP_001320930.1), *Caenorhabditis elegans* (NP_510144.1), *M. tuberculosis* H37Rv (NP_216726.1), *S. cerevisiae* S288C(NP_012078.3), *Schizosaccharomyces pombe* (NP_595180.2), *Hordeum vulgare* subsp. *vulgar*e (XP_5.044979232), *Triticum aestivum* (XP_044377163.1), *Danio rerio* (NP_956358.1), *Cucumis melo* (XP_008444507.1), *Mannheimia* sp. USDA-ARS-USMARC-1261 (AHG72724.1), *Streptococcus pneumoniae* (QUY39052.1), *Staphylococcus aureus* subsp. *aureus* ST228(CCJ14319.1), *Pseudomonas aeruginosa* BL05 (ERY42690.1), *Xenopus laevis* (XP_018106594.1), *Ovis aries* (NP_001009444.1), *Bos taurus* (NP_001077113.1), *Solanum tuberosum* (NP_001275385.1). The numbers represent the bootstrap values (in percentage) for each branch point.

Finding homologous sequences and analyzing their functions is one way to understand the general use of proteins. Subtle differences in gene sequences can have a significant impact on the function of protein. Comparing the gene sequences of transaminases from different species has a certain enlightening effect on the study of transaminases. The evolutionary laws of different species can be found in the evolution of transaminase genes. The BCAT protein sequences are downloaded from National Center for Biotechnology International. These files are downloaded in FASTA format. Transaminase sequences of different species obtained by NCBI were introduced through molecular evolutionary genetic analysis (MEGA) software. The Clustal algorithm is used for sequence alignment and the Neighbor joining method to build evolutionary trees (Saitou and Nei, 1987; Sievers and Higgins, 2021). Evolutionary trees infer their kinship based on the similarity of gene sequences, with closer branches meaning closer relatives, higher sequence similarities, and closer cumulative branch lengths indicating closer kinship (as shown in Figure 3).

## 3 BCAT gene expression and function in plants

BCAT1 in plants was recently studied in wheat (*Triticum aestivum*), where it was found that BCAT can regulate susceptibility to wheat rust (Corredor-Moreno et al., 2021). BCAA aminotransferase (known as TaBCAT1) was found in pathogen-infected bread wheat as a positive regulator of wheat rust sensitivity. Destruction of TaBCAT1 inhibited the infection of yellow rust and stem rust and increased levels of BCAA in TaBCAT1-destroyed mutants that regulated BCAA metabolism in wheat. TaBCAT1 mutants also showed elevated levels of salicylic acid and enhanced expression of related defense genes. There is a correlation between BCAA levels in wheat and yellow rust, highlighting the role of BCAT in plant defense responses. Disrupting *TaBCAT1* inhibits wheat infection by the wheat yellow and stem rust fungi, considering the crucial role of T*aBCAT1* in pathogen invasion, may be a target for manipulation to increase resistance to pathogen infection. The BCAT gene isolated in *H. vulgare* is regulated by drought stress. The transcription level of Hvbcat-1 increases under drought stress, and degradation of BCAAs can serve as a detoxification mechanism to keep free branched-chain amino acids at low or non-toxic levels (Malatrasi et al., 2006). BCAT can also improve the drought tolerance of rice (*Oryza sativa*), identifying cytosolic BCAT1 as a candidate gene for plant drought tolerance enhancement and participating in the accumulation of drought-stress-mediated BCAAs in rice (Shim et al., 2023). Overexpression of OsDAT under drought conditions in paddy fields increases cereal yields. Melon *BCAT1* gene was expressed in *Escherichia coli*, showing branched-chain amino acid transaminase activity. The expression of *BCAT1* is low in vegetative tissues, but during fruit ripening, expression in pulp and peel tissues increases. Ripe fruits of aromatic varieties generally show high expression of *BCAT1* compared to unripe fruits (Gonda et al., 2010). In tomatoes, the effects of monoisoform BCAT on fruits were studied. They found ketoacids rather than amino acids to be possible precursors of branched-chain flavor volatiles. The change in expression level of BCAT isoforms did not affect the synthesis of branched-chain volatiles, indicating that BCAT was not a suitable target for metabolic engineering of important flavor compounds (Kochevenko et al., 2012). It was found that the increase of BCAT gene expression induced by low temperature stress could promote the synthesis of Leu in tomato fruit (Zhang W. F. et al., 2019). BCAT is also potentially involved in drought-resistant pathways in *Arabidopsis*, where BCAT2 is a pathogenic gene with naturally variable BCAA content (Angelovici et al., 2013). BCATs are essential for the free growth of betarhizobia and their ability to form effective symbionts with host plant of the *Mimosa* genus (Chen et al., 2012). Plants have different levels of gene expression in different environments. The development of genome transcriptome sequencing has helped to understand plant defense responses in different environments and plant susceptibility to disease. A deeper understanding of BCAT will provide better protection for cash crops from pathogens and harsh environmental stresses.

## 4 BCAT gene expression and function in animals

Recent studies of BCAT1 in animals were conducted in mouse embryonic stem cells (Chen et al., 2020). Regulation of self-renewal and pluripotency of mouse embryonic stem cells is done by Ras signaling. High-throughput sequencing of undifferentiated mouse pluripotent stem cells as well as differentiated cells revealed that BCAT1 is highly expressed in mouse embryonic stem cells and is significantly downregulated in differentiated cells. The *BCAT1* gene in mouse embryonic stem cells was knocked out with CRISPR-cas9, which led to spontaneous differentiation of mouse embryonic stem cells. The specific mechanism of self-differentiation of mouse embryonic stem cells needs to be further studied. Genome-wide bisulfite sequencing (WGBS) analysis showed that BCAT1-knockout cells had reduced levels of DNA methylation on each chromosome throughout the genome, altering gene expression in multiple pathways. Further research into the function of BCAT1 in mouse embryonic stem cell (ESC) and the analysis of its underlying mechanisms is needed. BCAA aminotransferases (BCAT1/ECA39) are also involved in apoptosis. Overexpression of BCAT1/ECA39 in murine cells had no significant effect on the proliferation of cells grown at high serum concentrations, but cell viability decreased under conditions of serum deprivation (Eden and Benvenisty, 1999). Mice with deletion of mitochondrial branched-chain aminotransferase (BCATm) (expressed in muscle and brain glial cells) showed a significant increase in BCAAs. Loss of BCAA transamination leads to changes in dietary choices and hypothalamic neuropeptide gene expression (Purpera et al., 2012). The expression of the BCAT2 gene is regulated during mouse adipocyte differentiation and is affected by the level of nutrients (Kitsy et al., 2014). Alzheimer’s disease (AD) is a chronic neurodegenerative disease and the expression of BCAT1 was significantly reduced in mice with this disease (Li et al., 2018).

The nonsense mutation in the mouse BCAT2 gene can lead to BCAA catabolic defects and high levels of BCAAs accumulation in plasma and urine, the direct proarrhythmic effect of elevated BCAAs levels, and the mTOR pathway can eliminate the proarrhythmic effect caused by elevated BCAAs (Portero et al., 2022). BCAA catabolic defects lead to the accumulation of BCKAs in myocardia. BCKAs may promote heart failure by inhibiting the respiratory chain and increasing the superoxide production of mitochondria. On the contrary, enhancing the activity of branch-chain α-ketoate dehydrogenase can significantly reduce cardiac dysfunction after pressure overload (Sun et al., 2016). Mice with BCAT2 exon 4-6 targeting deletion showed lower body weight and exercise intolerance. BCAT1 inhibitors alleviate childhood asthma in mice by affecting airway remodeling and autophagy. BCAT1 is upregulated in chronic airway disease; however, its role in childhood asthma is unclear. BCAT1 is upregulated in mice with neonatal asthma, and BCAT1 inhibitors may inhibit airway inflammation and remodeling by reducing autophagy, which may provide a new therapeutic direction for childhood asthma (Li et al., 2022).

*Celetodes* parasitizing plants and animals have also been studied on BCAT1 (Mansfeld et al., 2015). Impaired BCAT1 gene expression in nematodes leads to increased lifespan and increased BCAA levels. Overexpression of BCAT1 impairs longevity and fecundity, and the transcription factor HLH-15 controls and effectively co-regulates physiological ageing with BCAT1. Consistent with the findings in rodents, supplementation with BCAAs extended the lifespan of nematodes. BCAAs act as peripherally derived metabolic factors that induce central nervous system endocrine responses that ultimately lead to a prolonged healthy range. Polymorphisms in the BCAT1 gene also have a protective effect against acute coronary syndrome (Ramírez-Bello et al., 2020). BCAT1 redox function maintains cell mitosis, which is essential for chromosome separation, and provides an explanation for BCAT1’s role in promoting cancer cell proliferation (Francois et al., 2022).

## 5 BCAT gene expression and function in microorganisms

BCAT in microorganisms has been widely studied, but BCAT purification has been less reported, and BCATs can be purified by column chromatography in *Helicobacter pylori* (Saito et al., 2007). Bacterial and archaeal BCAT is distinguished from eukaryotes by broad substrate specificity (Bezsudnova et al., 2017). Hyperthermophilic archaea exhibit high specificity for BCAAs and their ketone analogues (Bezsudnova et al., 2016). BCAT has been used in the biosynthesis of unnatural amino acids. Aminotransferases are important biocatalysts for the synthesis of chiral amines, have the ability to introduce amino groups into ketones or ketoacids, and have high enantioselectivity (Zheng et al., 2019). Most of them are introduced as branched-chain amino acids inserted into *E. coli* for clonal expression and purification (Stekhanova et al., 2017). *E. coli* BCAT can be used to synthesize some asymmetric unnatural amino acids (Yu et al., 2014), such as L-n-Leucine, L-n-Valine, and L-Neopentylglycine. The expression of BCAT in *E. coli* is associated with the production of pantothenic acid in industry (Zhang B. et al., 2019). It is expressed in *E. coli* by cloning BCAT from *Pseudomonas* (PsBCAT). PsBCAT shows a relatively wide substrate spectrum and exhibits significant activity against a large number of aliphatic L-amino acids. In addition, PsBCAT demonstrates activity with aromatic L-amino acids, such as L-histidine and L-threonine. The BCAT sequences of *Bacillus subtilis*, *Corynebacterium* glutamate, and *Pseudomonas* showed varying degrees of similarity with *E. coli*, respectively. BCAT protein is mainly produced in the form of a soluble protein, and the expression of PsBCAT is preferred over other BCATs and is more selective for substrates. So it is more likely to be widely used in industry.

The BCAT, encoded by the ilvE gene, is involved in acid tolerance of *Streptococcus* mutans, which is considered a pathogen of human dental caries and affects dental health as well as oral microbial survival. Under acid-stressed conditions, ilvE genes are upregulated in *Streptococcus* mutans, and growth lags in *Streptococcus* mutans ilvE mutant strains when nutrition restricts branched-chain amino acids (Santiago et al., 2012). In staphylococci, ilvE mutants were shown to lose enzymatic activity against BCAAs, demonstrating their role in amino acid catabolism (Madsen et al., 2002). Mutant strains of *Staphylococcus* carnosus lacking BCAT activity are unable to produce suitable branched-chain α ketoacid precursors for branched-chain fatty acid biosynthesis and need to be grown in medium containing 2-methylpropionic acid (Beck, 2005). *Mycobacterium* BCATs are key enzymes for the synthesis of methionine, and blocking methionine biosynthesis in mycobacteria can inhibit the growth of mycobacteria (Pham et al., 2022). In contrast to higher fungi, the biosynthetic genes of the oil-producing fungus *Mortierella alpina* BCAAs are virtually immune to transcriptional regulation, indicating a sustained production of BCAAs (Sonnabend et al., 2022). Isolation of *Lactobacillus fermentum* YZU-06 with high utilization rate of branched-chain amino acids in Jinhua ham is a promising starter culture that can improve the flavor of fermented meat products (Liu et al., 2022a). Moreover, the addition of Leu and the mixed starter of *L. fermentum* YZU-06 and *S. saprophyticus* CGMCC 3475 could produce methyl branched-chain aldehyde and improve the overall quality of fermented sausages (Liu et al., 2022b). *S. cerevisiae* BCATs are not only involved in the production of BCAAs but are also associated with the production of fusels as a precursor to volatile flavor components of next-generation biofuels or as fermentation products. The overexpression of these BCATs also increases the industrial production of fusel alcohols (Eden et al., 1996). Mitochondrial Bat1 in *S. cerevisiae* regulates Val biosynthesis, but Bat2 has little effect on Val biosynthesis. Whether the biosynthesis of Leuand Ile is mainly regulated by Bat1 or Bat2, or by both enzymes, has not been clearly explained. *S. cerevisiae* may also contain more than these two aminotransferases, which may be regulated by other undiscovered aminotransferases (Takpho et al., 2018).

Pathogens rely on host amino acids to grow and survive, BCATs regulate the metabolism of BCAAs, and BCAAs are essential for the growth of various microorganisms. Targeting BCATs may disrupt pathogen utilization of BCAAs, impairing their ability to reproduce. The development of BCAT inhibitors as potential antimicrobials can selectively block BCAT activity, such as Aminooxy compounds being detected as potential BCAT inhibitors, hindering pathogen utilization of BCAAs for growth and survival (Venos et al., 2004), but further research is needed to elucidate the specific mechanisms by which BCATs promote pathogen growth.

## 6 BCAAs aminotransferases and human diseases

BCATs are closely related to diabetes. Plasma BCAAs are significantly elevated in patients with type 1 diabetes mellitus (Karusheva et al., 2021), the decrease in glycolysis reduces the content of amino receptors (α-ketoglutarate, pyruvate, and oxaloacetate), the branched-chain amino acid transamination reaction is reduced, and the level of branched-chain amino acids in human plasma will be significantly increased, and the flux of BCAAs through BCKAD is impaired due to excess NADH and an increase in the ratio of acyl-CoA to CoA-SH (Holeček, 2020). In addition, BCAT1 methylation was found to be associated with obesity (Kaufman et al., 2018), elevated plasma BCAAs for obesity states (She et al., 2007), BCAT1 as a candidate gene associated with obesity (Chen et al., 2013), and obesity associated with type 2 diabetes (Nadler et al., 2000). BCAT1 single nucleotide polymorphisms were identified as associated with type 2 diabetes from a genome-wide association scans (Rampersaud et al., 2007), and in the skeletal muscle of patients with type 2 diabetes, the expression of the gene encoding the first step enzyme involved in BCAA metabolism (BCAT2) is reduced, and the patient’s branched-chain amino acid metabolism is inhibited. Glucose load further attenuates BCAA catabolism in patients with type 2 diabetes, and circulating plasma BCAA levels change. However, the transcriptome regulatory mechanisms of genes involved in BCAA catabolism are unknown (Sjögren et al., 2021).

BCATs are mainly expressed in the brain and neurons, and BCATs are involved in N shuttles between astrocytes and neurons in the brain, BCATs are closely related to brain diseases as well as neurological diseases (Knerr et al., 2019). Maple syrup urine disease (MSUD) is an inherited metabolic disorder, BCKD complex subunit mutations lead to the accumulation of BCKAs, the exact molecular mechanism by which BCKAs cause neuronal damage in MSUD is not fully understood, studies indicate that the accumulation of BCKAs disrupts mitochondrial function, induces oxidative stress, and impairs neurotransmitter synthesis and release (Funchal et al., 2006). Mitochondrial BCAT2 mutations in N-ethyl-N-nitrosourea-treated mices produce pathological features similar to MSUD, providing an animal model for studying the metabolism of BCAAs (Wu et al., 2004), unlike MSUD, elevated plasma BCAAs in patients with BCAT2 deficiency do not lead to acute encephalopathy (Knerr et al., 2019). Secondly, according to a study of Alzheimer’s patients, high levels of serum BCAA, glutamate, and BCAT were positively correlated with AD severity. Glutamate production is increased by elevated BCAT activity, which may deteriorate brain function and be associated with impaired cognitive function and faster cognitive decline (Hudd et al., 2019). BCATs participate in regulating the levels of neurotransmitters such as glutamate and γ-aminobutyric acid. BCAT inhibition may lead to an imbalance in neurotransmitter levels, leading to neurological diseases and neuronal dysfunction. The molecular mechanisms underlying BCAT dysfunction leading to these diseases include impaired neurotransmitter synthesis, mitochondrial dysfunction, and oxidative stress (Funchal et al., 2006). In the brains of patients with vascular dementia (VaD) and dementia with Lewy bodies (DLB), hBCATm protein expression was significantly increased, similar to that reported in AD brains (Ashby et al., 2017). Associated with Pap syndrome, a metabolic neuromuscular disease, overexpression of the BCAT gene can rescue the growth of cells lacking the phosphatidyltransferase TAZ1 gene (Antunes et al., 2019), The main contribution of BCATs may be the compensatory increase in TCA circulating flux, but the specific mechanism of the overexpression of the BCAT gene to rescue the growth of cells lacking the phosphatidyltransferase TAZ1 gene is unclear.

Overexpression of BCAT1 in leukaemia stem cells reduces intracellular α-ketoglutarate (αKG), and DNA demethylase inactivation leads to DNA hypermethylation (Raffel et al., 2017). BCAT1 regulates gene epigenetics by restricting intracellular αKG to stabilize the HIF1α protein required for the maintenance of leukemia stem cells (Raffel et al., 2017). In chronic myeloid leukaemia (CML), BCAT1 promotes clonal growth by forming BCAAs from amino groups of BCKAs. Conversely, reducing the expression of BCAT1 promotes cell differentiation and prevents the spread of CML *in vivo* (Hattori et al., 2017). BCAT has also been studied in cardiovascular diseases. BCAA aminotransferase 1-23C/G polymorphisms have been found to have a protective effect against acute coronary syndrome, but the specific mechanism has not been studied (Ramírez-Bello et al., 2020). Activation of BCAT1 and inhibition of oxoeicosanoid receptor can reduce acute myocardial infarction (Lai et al., 2021). Activation of BCAT1 can also reduce acute myocardial infarction; overexpression of BCAT in the heart can improve myocardial ischemic injury; and targeted treatment of BCAT is a promising strategy for acute myocardial infarction for clinical treatment (Lai et al., 2021). Increased BCAT activity in combination with the branched-chain α-ketoacid dehydrogenase (BCKD) kinase inhibitor BT2, which reduces cardiac BCAA levels, increased forward transamination rates and increased the therapeutic benefit of BCAAs (Voronova et al., 2022).

Most research on BCAT is related to cancer (details are shown in Table 3). BCAT1 shows different activities in cancer, correlated with tumor aggressiveness (Tönjes et al., 2013). BCAT1 is upregulated in isocitrate dehydrogenase (IDH) wildtype but not mutant glioblastoma multiforme (GBM) (Tönjes et al., 2013). The BCAT1 inhibitors gabapentin and α-ketoglutaric acid (αKG) kill wild-type IDH GBM cells. The principle is that the inhibition of BCAT1 increases the NAD^+^/NADH ratio with BCAT1 deletion, impairing oxidative phosphorylation, mTORC1 activity, and nucleotide biosynthesis, leading to mitochondrial dysfunction and loss of ATP, nucleotides, and proteins. (Zhang B. et al., 2022). The hypermethylation of the BCAT1 promoter inhibits the BCAT1 gene, and the decrease in the expression of BCAT1 reduces the supply of glutamate, increases the dependence on glutaminase, and reduces tumor proliferation and invasion (Yi et al., 2021). High expression of BCAT1 is a marker for predicting poor prognosis in IDH1 wild-type glioma patients, providing an important target site for glioblastoma patients (Yi et al., 2021; Huang W. et al., 2022). Noncoding RNAs have been widely studied in recent years, glioblastoma is also regulated by competitive endogenous RNA, circVPS18 accelerates the progression of glioblastoma through miR-1229-3p/BCAT1, providing a potential therapeutic target for glioblastoma (Huang Q. et al., 2022).

**TABLE 3:** BCAT expression and related studies in different types of cancer.

Cancer type	BCAT expression	Description of relevant studies	References
Glioblastoma	High BCAT1 expression in IDH^wt^ but was essentially absent in IDH^mut^	BCAT1 deletion combined with αKG is a new synthetic lethal method for the treatment of IDH^wt^ GBM. The decrease in the expression of BCAT1 reduces the supply of glutamate, increases the dependence on glutaminase, and reduces tumor proliferation and invasion	Tönjes et al. (2013), Yi et al. (2021)
Liver cancer	High BCAT1 expression in HCC.	BCAT1 promotes HCC cell development and metastasis by activating the AKT signaling pathway and epithelial-mesenchymal transformation (EMT)	Ding et al. (2023)
Breast cancer	High BCAT1 expression in Breast cancer	BCAT1 knockdown may help reduce mTOR signaling and reduce the growth rate of breast cancer cell lines	Zhang and Han (2017)
Prostate Cancer	Low levels of BCAT activity	BCAT levels were elevated in healthy human prostate tissue relative to malignant tissue	Billingsley et al. (2014)
Colorectal Cancer	High BCAT1 expression in Colorectal Cancer	Long non-coding RNA (lncRNA) TMPO-AS1 can upregulate the expression of BCAT1 through miR-98-5p, promoting the progression of colorectal cancer cells	Xu et al. (2021), Ye et al. (2022)
Pancreatic ductal adenocarcinoma (PDAC)	High BCAT2 expression in PDAC.	BCAT2 knockdown significantly inhibited the proliferation of PDAC cells, reduced cellular fatty acid levels and associated with lipid synthesis	Lee et al. (2019)
Pancreatic cancer	High BCAT2 expression in Pancreatic cancer	Acetylation promotes BCAT2 degradation to suppress BCAA catabolism and pancreatic cancer growth	Lei et al. (2020)
Gastric cancer	Low BCAT2 expression in Gastric cancer	Dysregulation of BCAT2 correlates with the overall survival time of gastric cancer patients	Zhang et al. (2022b)
Non-small cell lung cancer	High BCAT1 expression in Non-small cell lung cancer	BCAT1 promotes cell proliferation and invasion by regulating the Wnt signaling pathway	Lin et al. (2020)

Expression and methylation of the BCAT1 gene are associated with human nonalcoholic fatty liver disease. BCAT1 may identify patients at risk for a poor prognosis (Wegermann et al., 2018). Tumors in patients with liver cancer (HCC) show abnormally high expression of the BCAT gene but low expression of BCKD and downstream catabolic enzymes (Ericksen et al., 2019). BCAT1 promotes HCC cell development and metastasis by activating the AKT signaling pathway and epithelial-mesenchymal transformation (EMT) (Ding et al., 2023). High concentrations of gabapentin can inhibit cell proliferation without affecting BCAT1 (Grankvist et al., 2018). BCATc inhibitor 2 is an inhibitor of cytosolic BCAT, which can be synthesized by binding 2-CF_3_-phenyls to coumarin derivatives (Lu et al., 2022). The cytosolic branched-chain amino acid aminotransferase inhibitor BCATc Inhibitor 2, which protects oleic acid-induced lipid accumulation and apoptosis, is a promising candidate for the treatment of Non-alcoholic fatty liver disease (Lu et al., 2022). Elevated BCAA levels promote excessive activation of mTOR signaling to promote tumor growth. Decreased levels of BCAAs in hepatoma cell culture medium reduce the proliferation rate of hepatoma cells (Ericksen et al., 2019). High expression of BCAT1 and BCKD genes in breast cancer activates mTOR signaling, and BCAA catabolism is enhanced. BCAT1 knockdown may help reduce mTOR signaling and reduce the growth rate of breast cancer cell lines (Zhang and Han, 2017). These studies have found a major role in tumor proliferation. In addition, in the presence of cachexia disease in cancer patients, supplementation with BCAAs improves quality of life due to their anabolic effects and subsequently increases the effectiveness of chemotherapy interventions (Laviano et al., 2005). The tumor diameter in the high-expression group was higher than that in the low-expression group, and the 5-year survival rate was lower in the high-expression group. The high expression of BCAT1 was positively correlated with tumor diameter, lymphatic metastasis, and a poor prognosis. BCAT1 is a potential biological target for breast cancer (Laviano et al., 2005). Another study was to silence the *BCAT2* gene in primary cells of the breast cancer cell line MCF-7 and breast tumor to reduce cell proliferation, but the detailed mechanism needs further investigation (Antanavičiūtė et al., 2017). Spectrophotometry and hyperpolarized 13C magnetic resonance spectroscopy (MRS) determined BCAT enzyme activity *in vitro* for prostate cancer of various origins (humans, mice). Low levels of BCAT activity were present in all prostate cancer models. And BCAT levels were elevated in healthy human prostate tissue relative to malignant tissue (Billingsley et al., 2014). N6-methyladenosine (m6A) RNA methylation plays an important role in tumorigenesis and metastasis, BCAT1 was screened for m6A RNA methylation-related gene in pancreatic cancer, which may be a new therapeutic target, but detailed mechanistic studies have yet to be discovered (Geng et al., 2020). BCAT1 is abnormally expressed in colorectal cancer (CRC) with significantly higher levels of cyclic tumor DNA (ctDNA) methylation, which can be used for CRC diagnosis with high sensitivity and specificity (Xu et al., 2021). Moreover, it was found that long non-coding RNA (lncRNA) TMPO-AS1 can upregulate the expression of BCAT1 through miR-98-5p, thereby promoting the progression of colorectal cancer cells (Ye et al., 2022). BCAT2 protein levels were significantly elevated in human pancreatic ductal adenocarcinoma (PDAC) cells, and BCAT2 knock down significantly inhibited the proliferation of PDAC cells. BCAT2 knockdown significantly inhibited the proliferation of PDAC cells, but untransformed human pancreatic ductal HPDE cells did not, and reduced cellular fatty acid levels were associated with lipid synthesis (Lee et al., 2019). Acetylating lysine 44 (K44), an evolutionarily relatively conserved residue located at the N-terminus of BCAT2, of BCAT2 promotes BCAT2 degradation and inhibits BCAA catabolism and pancreatic cancer growth (Lei et al., 2020). Therefore, BCAT2 may also provide a new therapeutic target for pancreatic cancer, presumably use of the modified BGAT may help the treatment of cancer. In addition, dysregulation of BCAT2 correlates with the overall survival time of gastric cancer patients, and this study contributes to the potential treatment of gastric cancer (Zhang Y. et al., 2022). BCAT1 was found to be a potential therapeutic target for clinical treatment of lung diseases. BCAT1 is overexpressed in human non-small cell lung cancer, and BCAT1 promotes cell proliferation and invasion by regulating the Wnt signaling pathway (Lin et al., 2020). In addition, abnormalities in smooth muscle cells of the pulmonary artery are associated with autophagy, and BCAT1 binds to IRE1 in the endoplasmic reticulum to activate the expression of its downstream pathway XBP-1-RIDD axis, thereby activating autophagy. The RNA-binding protein zinc finger protein 423 promotes autophagy by binding to BCAT1mRNA 3′-untranslated regions (Xin et al., 2020).

BCAT2 can also be used to predict cancer cell responsiveness to ferroptosis-inducing therapies and be used as a sensitive biomarker to evaluate drug response in preclinical cancer models (Wang et al., 2021). BCAT2 plays a crucial role in ferroptosis. Ferroptosis inducers (erastin, sorafenib, and sulfazazine) activate the AMPK/SREBP1 signaling pathway through iron-dependent ferritinophagy, inhibiting BCAT2 transcription (Wang et al., 2021). The overexpression of *BCAT2* in the human pancreatic cancer cell line Aspc-1 and hepatocellular carcinoma cells HepG2 increased intracellular glutamate and glutamate release, increased system Xc^−^ activity, and inhibited ferroptosis. System Xc^−^ is located in the cell membrane and mediates cysteine and glutamate transport (Liu et al., 2021). Inhibition of the expression of the BCAT2 gene by RNA interference, thus blocking system Xc^−^ activity, resulted in ferroptosis in cells. Thus, knockdown of the expression of the BCAT2 gene can partially induce ferroptosis. Therefore, highly specific BCAT2 inhibitors can provide an effective treatment for a subset of cancer patients. Conversely, BCAT1 activity is significantly reduced in ferroptosis-induced mesenchymal stem cells, which downregulate ferroptosis by inhibiting transcription of glutathione peroxidase-4 (GPX4) in mesenchymal stem cells (Hu et al., 2023). In different ferroptosis-induced cells, the activity of branched-chain amino acid aminotransferases of different subtypes is altered, and further research is needed.

BCAT converts BCAA to BCKA, which can be further metabolized by the branched-chain ketoacid dehydrogenase (BCKDH) complex, producing acetyl-CoA and entering the tricarboxylic acid (TCA) cycle, providing an additional source of energy for cancer cells (Mayers and Vander Heiden, 2017). In addition, BCAT plays a role in supporting cancer cell growth and proliferation by promoting the biosynthesis of key molecules required for tumor development, such as activating or inhibiting the mTOR signaling pathway to affect cancer cell multiplication (Wolfson et al., 2016; Zhang and Han, 2017). BCKAs produced by BCAT can serve as precursors for the synthesis of fatty acids, cholesterol and other important metabolites. The BCAT-catalyzed transamination reaction leads to the formation of glutamate, which can be used for the biosynthesis of other non-essential amino acids such as glutamine, or the production of a-ketoglutaric acid by other aminotransferases (Altman et al., 2016), followed by glutamic acid as a precursor to the synthesis of glutathione, a major antioxidant molecule. By promoting glutathione synthesis, BCAT helps cancer cells maintain a favorable intracellular redox environment that supports their survival and proliferation (Godwin et al., 1992).

## 7 Conclusion

In recent years, in-depth research on BCATs have been carried out, from humans to animals and plants and microorganisms, from BCATs chemical structure to function, from BCAT genes to related pathway studies, and have a clearer understanding of BCATs. Research on microbial BCAT is mainly related to the industrial production of its products. Since BCAAs are essential amino acids for humans and cannot be synthesized by humans, the industrial production of BCAAs is crucial. BCATs are not only involved in the production of BCAAs, but also related to the preparation of fusel alcohols and methyl branched-chain flavor compounds, so BCATs can be used to produce fuels and are widely used in the food industry. The study of BCAT will likely improve productivity as well as reduce costs, such as increased enzyme activity or substrate specificity. Plant BCATs are mostly studied on economic crops (barley, wheat, rice, etc.), and mainly emphasizes the role of BCATs in plant defense response, resistance to pathogen invasion and drought or low temperature stress, in-depth study of BCATs will benefit the cultivation of economic crops and food and fruit yield, and better benefit mankind. BCAT studies in animals are associated with diseases, most of which are caused by disorders of branched-chain amino acid metabolism, such as metabolic disorders, cancer, and neurodegenerative diseases. BCAAs metabolism is regulated by a variety of factors. Hormonal regulation (e.g., insulin, glucagon, and growth factors) can affect transcriptional regulation of BCAA pathway enzymes. In addition, multiple signal modulations such as mTOR signaling, insulin signaling, and AMPK signaling. Enzyme regulation, BCKDC kinase (BCKDK) with protein phosphatase 2Cm (PP2Cm) alters branched-chain α-ketoacid dehydrogenase complex (BCKDC) activity to regulate BCAA catabolism. Non-coding RNAs (miRNA, circRNA, lncRNA) are involved in the post-transcriptional regulation of BCAT. Current research on BCAT has certain limitations, such as measuring enzyme activity *in vitro*, it is impossible to completely mimic the *in vivo* environment, and most of the research on diseases only shows its correlation, without in-depth study of molecular mechanisms. In-depth understanding of the relationship between BCAT and its gene expression, and the use of genetic engineering to change its activity may provide more accurate and effective treatment. BCAT is not limited to catalysis, but more importantly, it regulates metabolism in the body. Metabolic disorders will inevitably cause a variety of diseases, and further research on its regulatory mechanism may provide a new direction for disease treatment. Cancer has become the main cause of death in humans, *in vitro* research on cancer cells is limited, after all, the metabolic pathways of the human body *in vivo* are complex and interrelated, BCAT is an important prognostic tumor marker, the development of drugs for BCAT, will provide new treatment strategies.
